# Biochar Loaded with a Bacterial Strain N33 Facilitates Pecan Seedling Growth and Shapes Rhizosphere Microbial Community

**DOI:** 10.3390/plants13091226

**Published:** 2024-04-28

**Authors:** Zexuan Jiang, Qi Li, Fangren Peng, Jinping Yu

**Affiliations:** 1 College of Forestry and Grassland, College of Soil and Water Conservation, Nanjing Forestry University, Nanjing 210037, China; m17354973215@163.com; 2 Jiangsu Key Laboratory for the Research and Utilization of Plant Resources, Institute of Botany, Jiangsu Province and Chinese Academy of Sciences (Nanjing Botanical Garden Mem. Sun Yat-Sen), Nanjing 210014, China; liqi@cnbg.net

**Keywords:** biochar, microbe, *Carya illinoinensis*, plant growth, microbial community

## Abstract

Biochar and beneficial microorganisms have been widely used in ecological agriculture. However, the impact of biochar loaded with microbes (BM) on plant growth remains to be understood. In this study, BM was produced by incubating pecan biochar with the bacterial strain N33, and the effects of BM on pecan growth and the microbial community in the rhizosphere were explored. BM application significantly enhanced the biomass and height of pecan plants. Meanwhile, BM treatment improved nutrient uptake in plants and significantly increased the chlorophyll, soluble sugars, and soluble proteins of plants. Furthermore, BM treatment improved the soil texture and environment. Finally, BM application substantially enhanced the diversity of soil fungi and bacteria as well as the relative abundances of the phyla *Firmicutes* and *Chloroflexi*, and families *Bacillaceae* and *Paenibacillaceae*, as shown by high-throughput sequencing. Together, this study clarified the growth-promotive effects of BM on pecan plants and suggested an alternative to synthetic fertilizers in their production.

## 1. Introduction

Pecan, or *Carya illinoinensis*, a crucial nut tree, possesses significant ecological and economic benefits [1]. *C. illinoinensis* seedlings planted in containers grow well and have well-developed root systems, and the robustness of container seedlings is better than bare root seedlings, but most growers in China cannot afford them [2]. Moreover, container seedlings require high-quality soil conditions, rigorous water and fertilizer management, and a prolonged growth period [3]. These factors limit the large-scale cultivation of container pecan seedlings. The rhizosphere-colonizing plant growth-promoting rhizobacteria (PGPRs) can promote the growth of plants through several mechanisms. The rhizosphere of plants contains various rhizosphere bacteria possessing the potential to promote plant growth and other bioactivities [4]. PGPRs are considered the most crucial organisms that influence plants’ development and growth [5]. Hence, PGPRs are able to function as a biofertilizer through improving plants’ nutrient availability, as a biostimulator through secreting phytohormones crucial for plants’ growth, and as a biopesticide through antagonizing plant pathogens or fostering systemic resistance. However, unfavorable factors like genetic diversity, environmental conditions, and the impact of native microbial communities may, solely or in combination, result in decreased PGPR efficiency in field settings [6]. For instance, plant growth and root exudation are impacted both directly and indirectly by native microbial communities [7,8,9,10]. Therefore, inoculated PGPRs need to successfully proliferate, survive, and efficiently promote the growth of host plants [11]. However, reports of the impact of PGPRs on pecan seedlings’ growth are rare.

In recent years, as an organic alternative to synthetic fertilizers, biochar has attracted increasing attention and acts as a potential soil amendment [12]. Carbonaceous material or biochar is produced under relatively low pyrolysis temperatures and oxygen-limited conditions from a variety of biomasses. Because of its porous structure and vast surface area, biochar has been extensively used in soil improvement [13]. Applying biochar substantially reduces the bulk density (BD), adsorbs heavy metals from the soil, enhances the soil’s cation exchange capacity (CEC), and raises its water-holding capacity (WHC) [14]. It could efficaciously improve soil environments for cultivating crops and promote plant growth. Numerous research has shown that biochar could function as a reservoir of nutrients in soil [15]. So, we hypothesize that the large pore structure of biochar can offer an environment suitable for microorganisms, increasing the scope of survival and reducing predation risk.

It has recently been discovered that biochar is an appropriate carrier for bacteria that promote plant growth. It was reported that pine biochar loaded with *Enterobacter cloacae* UW5 in cucumber plants efficaciously reduced the soil BD, improved the soil WHC, and promoted the plants’ development and growth [16]. Moreover, a superior effect of biochar loaded with the *Bacillus subtilis* strain B38 was noted on immobilizing heavy metals in soil and promoting plants’ growth [17]. In comparison with peat alone, biochar loaded with *Bacillus cereus* significantly elevated the relative abundances of *Firmicutes*, *Actinobacteria*, *Proteobacteria*, and *Cyanobacteria* communities [18]. Therefore, biochar loaded with microorganisms can efficiently enhance the soil environment and facilitate plant growth. However, it is still unclear whether combining microbes and biochar could enhance pecan growth. Therefore, we plan to explore the effects of biochar loaded with microbes on pecan plants.

Plants live in close associations with a diversity of microorganisms. These microbes support plant health in various ways. For example, the phenomenon of volatile organic compounds (VOCs) bridging the mutualistic interactions between bacteria associated with plants and their hosts is frequently reported [19]. In addition, the potential of VOCs to suppress plant pathogens, including RKNs (root-knot nematodes), has gained increasing attention, suggesting a possible approach to managing pests and pathogens [20]. Meanwhile, root-secreted chemicals could affect multipartite interactions in the rhizosphere, and plant roots as well as their immediate environment continually respond to these chemicals. The diterpenoid metabolites of maize (*Zea mays*) displayed antifungal bioactivities and also influenced rhizosphere bacterial communities, facilitating the growth and well-being of plants [21]. However, the effects of biochar loaded with microbes on the rhizospheric soil microbes of pecans are still unknown.

## 2. Results

### 2.1. Loading Biochar with N33

We used the physical adsorption method to prepare biochar loaded with microbes (BM) [22]. In order to screen the most suitable N33 concentration, we set up five concentration gradients (Table 1). By measuring the number of biochar-supported bacteria and calculating the survival rate after loading the biochar with N33, we found that when the initial concentration of N33 suspension was 6.68 ± 0.62 × 10^5^ CFU/mL, the number of biochar-supported bacteria and survival rate were 2.83 ± 0.48 × 10^5^ CFU/mL and 84.6 ± 0.04%, respectively, which were higher than other treatments. Therefore, we chose this concentration to produce BM for the subsequent pot experiment. This result also indicated that biochar loaded with bacteria was highly efficient and the biochar could adsorb most of the bacteria in the bacterial suspensions in a very short period of time. The scanning electron microscope (SEM) images of the biochar and N33 are displayed in Figure S1A. As observed, the biochar alone showed a coarse surface and porous structures. The majority of the cells appeared dispersed or aggregated on the surface of the biochar. The results also showed that the strain N33 adhered well to the biochar.

### 2.2. BM Promoted Pecan Plants’ Growth

To explore the growth-promoting effects of N33 (microbe, M), biochar (B), and biochar loaded with N33 (biochar with microbes, BM), we conducted potting trials using pecan seedlings. First, we collected rhizospheric soil samples from pecan seedlings. The number of N33 in each soil sample was determined by the plate-counting method. The amount of N33 after the application of BM in rhizospheric soil was significantly larger than that following M treatment (Figure S1B,C). Therefore, the application of N33 in the form of biochar loaded with bacteria can improve its colonization in rhizospheric soil.

**Figure 1:**
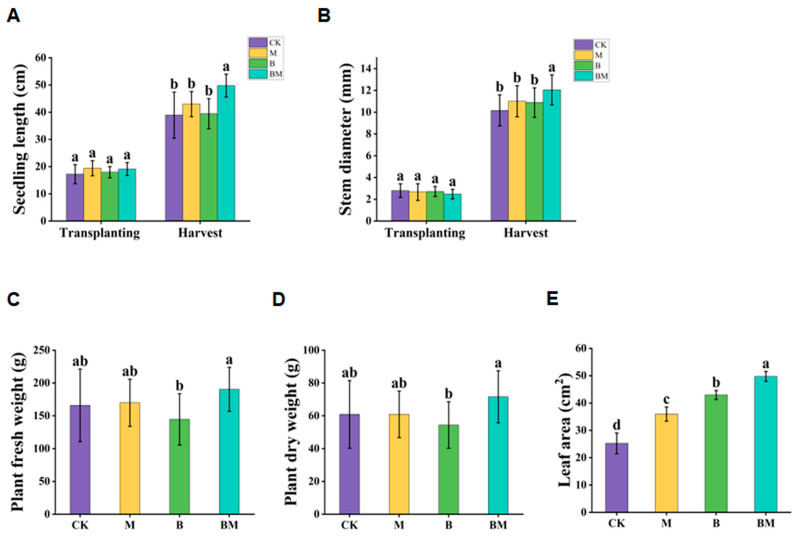
The growth indexes of pecan plants under different treatments. The seedling length (**A**), stem diameter (**B**), plant fresh weight (**C**), dry weight (**D**), and leaf area (**E**) of pecan plants were recorded. Data are shown as the means ± standard deviation of fifteen replicates; different lowercase letters indicate significant differences at *p* < 0.05.

Figure 1 displays the biomass and growth of pecan plants following various treatments. The stem diameter and height of pecan seedlings were measured after transplanting and at harvest, directly reflecting the plants’ growth under various treatments (Figure 1A,B). Among them, the seedling height and stem diameter of BM-treated plants showed a significant growth trend. The dry and fresh weights of pecan plants slightly increased under M treatment, in comparison with the CK. On the other hand, the BM treatment produced maximum biomass and substantially increased the dry and fresh weights of pecan plants, respectively, by 14.79% and 17.66% (Figure 1C,D). Moreover, in comparison with the CK, the leaf area under BM treatment increased by 74.43% (Figure 1E). Overall, we discovered that the BM treatment could substantially stimulate pecan plants’ growth.

### 2.3. Effects on Physiology of Pecan Plants

**Figure 2:**
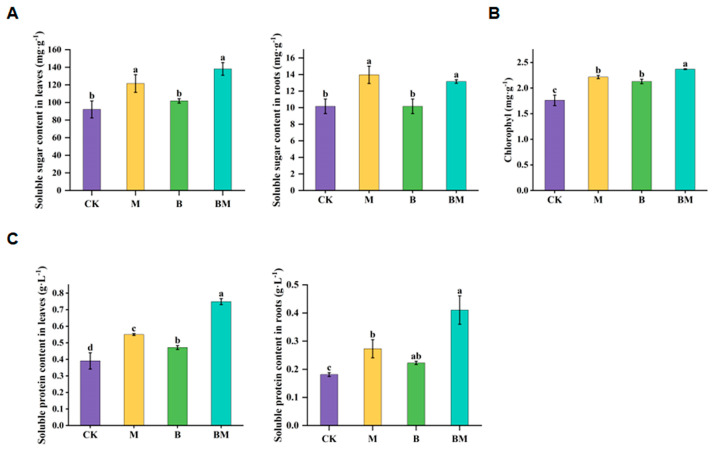
The physiology indexes of pecan plants under different treatments. Soluble sugar in roots and leaves (**A**), chlorophyll (**B**), and soluble protein in roots and leaves (**C**) were recorded. Data are shown as the means ± standard deviation of fifteen replicates; different lowercase letters indicate significant differences at *p* < 0.05.

To examine the effect of various treatments on inoculated plant physiology, we measured the levels of soluble sugar, soluble protein, chlorophyll, total nitrogen (TN), total phosphorus (TP), and total potassium (TK) of the plants. The contents of soluble sugar content in roots and leaves under any treatment were higher than the control; in particular, those under BM treatment were significantly higher than the control, with 29.5% and 49.92% increases, respectively (Figure 2A). According to Figure 2C, the content of soluble protein was increased in M, B, and BM treatments, compared to the control, but it was significantly increased in the BM treatment. As the major pigment in plants involved in photosynthesis, chlorophyll reflects the plant’s photosynthetic capacity and health [23], we examined the effect of various treatments on the chlorophyll of pecan plants. Relative to CK, the M, B, and BM treatments remarkably improved the contents of chlorophyll in leaves (Figure 2B). Among these treatments, BM treatment affected chlorophyll content in pecan plants most remarkably, enhancing the chlorophyll content by 34.6% (Figure 2B). Moreover, root vigor was significantly increased by 75.41% in BM treatment, compared to the control, whereas BM treatment did not remarkably affect the activities of peroxidase (POD) and superoxide dismutase (SOD) (Table 2).

**Figure 3:**
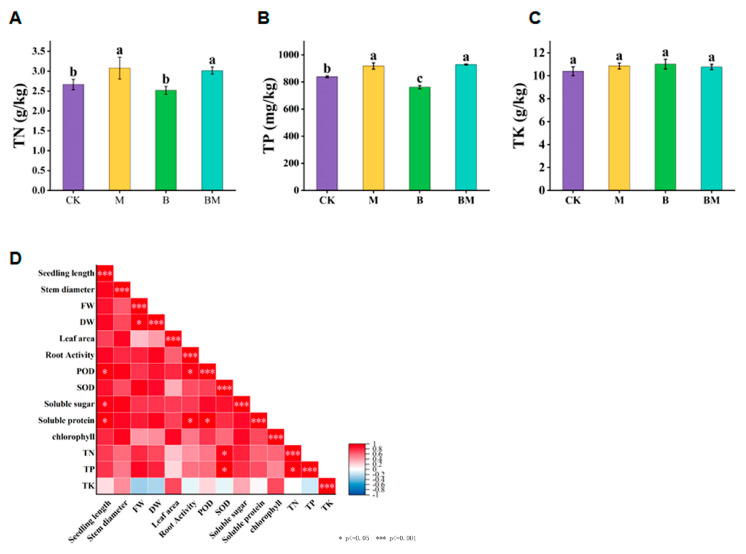
The physiology indexes of pecan plants under different treatments and correlation analysis. TN (**A**), TP (**B**), TK (**C**), and correlation analysis of growth and physiological indexes of pecan plants were recorded (**D**). Stars represent significant correlations. Color brightness indicates correlation coefficient values.

The contents of TN and TP of plants under M and BM treatments were higher than those under control at significant levels, but the content of TK in pecan plants did not change significantly in any treatment (Figure 3A–C). The contents of TN and TP in M-treated plants increased by 15.53% and 9.45%, respectively, while their contents in BM-treated plants increased by 13.18% and 10.72%, respectively (Figure 3A,B). In conclusion, the biochar loaded with bacteria promoted the nutrient absorption of pecan plants. Based on this, we conducted a correlation analysis for the growth indexes and physiological indexes (Figure 3D). Significant positive correlations were observed between seedling length, POD and SOD, soluble sugar, and soluble protein (Figure 3D). Soluble protein was positively correlated with POD and root vitality. Moreover, SOD positively correlated with TN and TP, while TP positively correlated with TN. These findings suggested that BM can increase photosynthesis and promote nutrient absorption, which results in plant growth stimulation.

### 2.4. Effects of Treatments on Soil Chemical and Physical Characteristics

**Figure 4:**
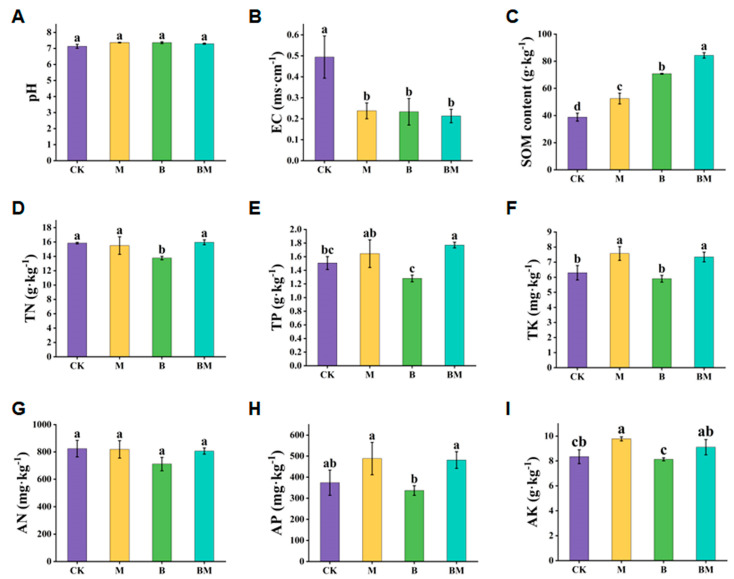
Effects of various treatments on soil chemical and physical characteristics in the rhizosphere of pecan plants. The pH (**A**), EC (**B**), SOM (**C**), TN (**D**), TP (**E**), TK (**F**), AN (**G**), AP (**H**), and TK (**I**) were recorded. All data are expressed in the form of mean (*n* = 3) ± standard deviation; different lowercase letters indicate significant differences at *p* < 0.05.

To investigate the effects of biochar loaded with bacteria on soil properties, we examined the chemical and physical characteristics of the soil under various treatments (Figure 4). Figure 4A shows that none of the treatments significantly altered soil pH, while all the other treatments notably reduced the soil electrical conductivity (EC) compared to the CK (Figure 4B). The soil organic matter (SOM) under the four treatments (CK, M, B, and BM) was significantly different from high to low (Figure 4C). As mentioned above, the soil contents of TN and TP in pecan plants under M and BM treatments were higher than those in the control group. However, the TK and TP contents instead of the TN under BM treatment were significantly higher relative to the control group (Figure 4D–F). The available soil N, P, and K contents under various treatments were also evaluated. Different treatment groups exhibited slight differences in the available N (AN) levels (Figure 4G). The M- and BM-treated soil had higher available P (AP) content than the control (Figure 4H); however, the available K (AK) level in M-treated soil was substantially higher than that in the control (Figure 4I). Therefore, the treatment of biochar loaded with bacteria can positively affect soil properties.

### 2.5. Effects of the Treatments on Soil Enzyme Activities

**Figure 5:**
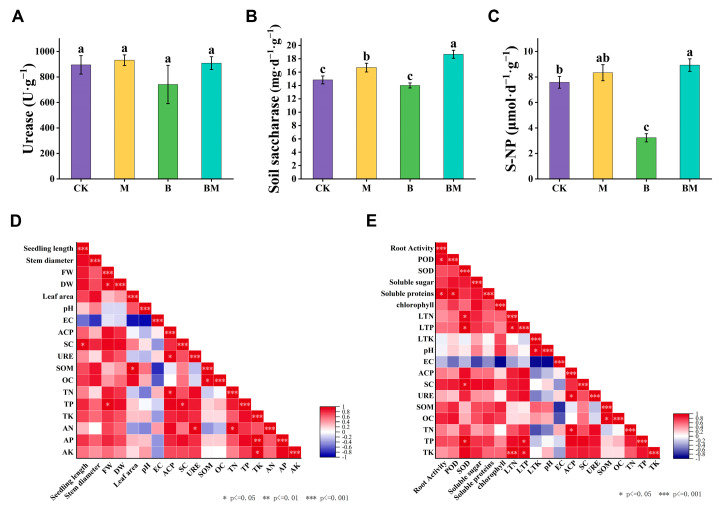
Effects of various treatments on soil enzyme activities and correlation analysis. Urease (**A**), soil saccharase (**B**), soil neutral phosphatase (S-NP) (**C**), and correlation analyses of pecan plant growth with soil physiological indexes (**D**) and physiological indexes with soil physiological indexes (**E**) were recorded. Stars represent significant correlations. Color brightness indicates correlation coefficient values.

To investigate the effects of biochar loaded with bacteria on soil fertility, we measured soil enzyme activities, including soil urease, saccharase, and neutral phosphatase (NP). As shown in Figure 5A, urease activity was slightly increased after M and BM applications relative to CK. However, saccharase activity of the rhizosphere soil under M and BM treatments was elevated as well, showing 12.41% and 25.89% increases in comparison to the control, respectively. In addition, the activity of neutral phosphatase increased significantly by 9.89% (M) and 17.78% (BM), respectively. (Figure 5B,C). These results proposed that the addition of bacteria and biochar loaded with bacteria could improve the enzymatic activities of the soil, resulting in stimulated growth of pecan plants. Furthermore, significant positive correlations were observed between seedling length and SC. A positive correlation was found between ACP and TN or URE, while a significant positive correlation was found between TK and AK or AP (Figure 5D). Significant positive correlations were also observed between SOD and TP, SC, LTN, and LTP. Root vitality was positively correlated with POD and soluble protein. Moreover, LTN correlated positively with TK and LTP, while ACP showed a correlation with TN and URE (Figure 5E).

### 2.6. Effects of Treatments on Soil Microbial Community and Composition

**Figure 6:**
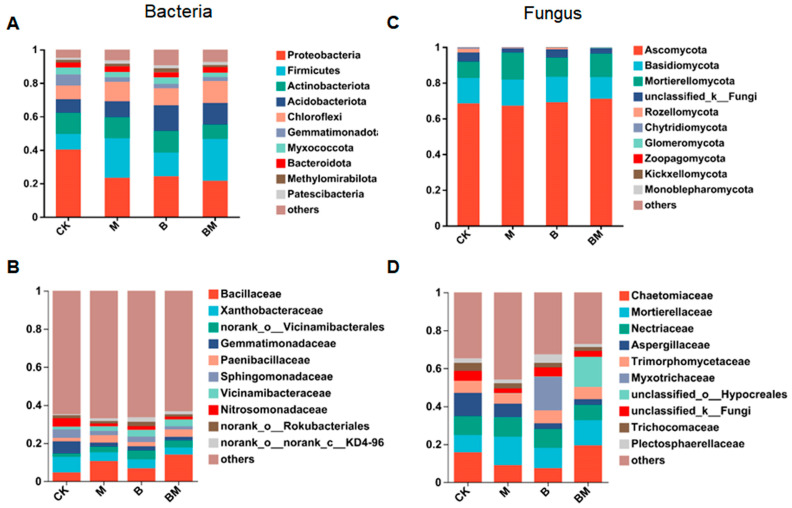
The rhizosphere microbial community composition varies under different treatments. Community composition of soil bacteria with top 10 relative abundances at the phylum (**A**) and family (**B**) levels. Community composition of soil fungi with top 10 relative abundances at the phylum (**C**) and family (**D**) levels.

The bacterial community structure in each treatment was examined by high-throughput sequencing to provide insights into the microbial differences in the rhizosphere soils of pecan plants. Figure 6A displays the relative bacterial phyla abundances. In comparison to CK, the relative soil *Proteobacteria* abundance remarkably decreased under M, B, and BM treatments. Biochar elevated the relative soil *Acidobacteriota* abundance. Figure 6A shows that M and BM treatments significantly elevated the relative *Firmicutes* abundance in comparison with the CK. In addition, they also substantially elevated the relative *Chloroflexi* abundance in comparison with that under B and CK treatments (Figure 6A). Throughout different treatments, 10 fungal phyla were detected. Relative to CK, the relative fungal abundances under each treatment were insignificantly changed (Figure 6C). Next, we checked the top 10 bacterial families in the soil (>0.1%). Relative to CK, treatment with M and BM substantially elevated the relative abundances of *Bacillaceae* and *Paenibacillaceae*, and that of *Bacillaceae* in the BM group was higher than under M treatment. Furthermore, relative to CK, treatments with M, B, and BM substantially reduced the abundance of bacterial families such as *Xanthobacteraceae*, *Gemmatimonadaceae,* and *Sphingomonadaceae* in the soil (Figure 6B). Finally, the top 10 soil fungal families were identified. As displayed in Figure 6D, all the other treatments elevated the *Mortierellaceae* abundance and reduced the *Aspergillaceae* abundance in comparison with the CK. The relative *Myxotrichaceae* abundance under treatment B increased dramatically, but other fungi did not exhibit any discernible changes under any of the other treatments (Figure 6D). These findings clearly show that post-treatment, the bacterial community varied, indicating that biochar, bacteria, and biochar loaded with bacteria treatment markedly affected the microbial community diversity.

**Figure 7:**
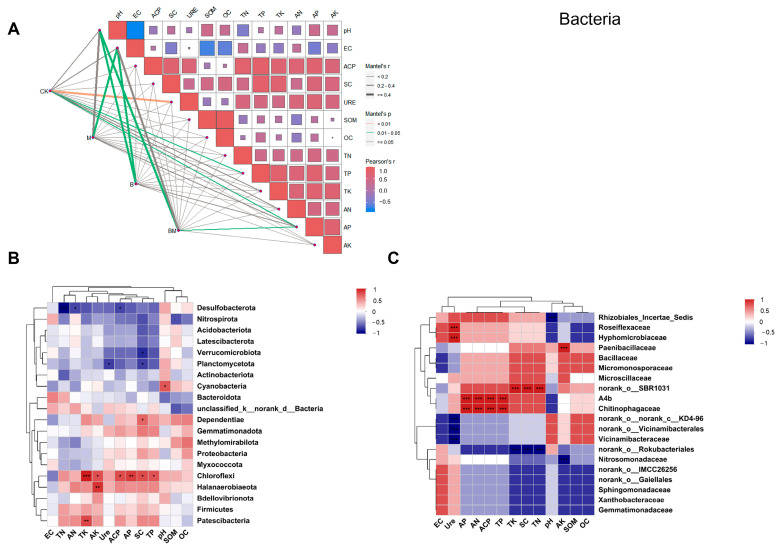
Relationships between the rhizosphere bacterial communities under various treatments and soil enzyme activity, as well as interactions between soil physicochemical parameters (**A**). Spearman correlation analyses for the physicochemical factors and the top 20 abundant bacteria at the phylum (**B**) and family (**C**) levels. Stars represent significant correlations (*p* < 0.01 for two stars, *p* < 0.05 for one star and *p* < 0.001 for three stars) of these indexes. Color brightness indicates correlation coefficient values.

Correlation heatmaps and the Mantel test network were employed to examine the link between the rhizosphere microbial community structure and soil physicochemical parameters under various treatments. In terms of rhizosphere bacterial communities, a strong correlation was noted with soil physicochemical properties and rhizospheric soil enzymatic activities. The correlation of pH and EC with soil nutrient indexes was negative (Figure 7A). At the phylum level of bacteria, soil physicochemical properties and rhizosphere bacterial communities exhibited an irregular trend among different treatments. All four treatments were significantly influenced by pH and EC. Furthermore, except for SOM and OC, significant correlations were noted with all nutrients. The correlation heatmap displayed that *Chloroflexi* was highly significantly and positively correlated with TK, AK, ACP, AP, SC, and TP, while *Desulfobacterota* was highly significantly and negatively associated with TN, AN, and ACP (Figure 7B). At the family level, bacteria *A4b* and *Chitinophagaceae* were highly, significantly, and positively associated with AP, AN, ACP, and TP. *Roseiflexaceae* and *Hyphomicrobiaceae* showed highly significant and positive correlations with URE. However, *Vicinamibacteraceae* showed highly significant and negative correlations with URE (Figure 7C).

**Figure 8:**
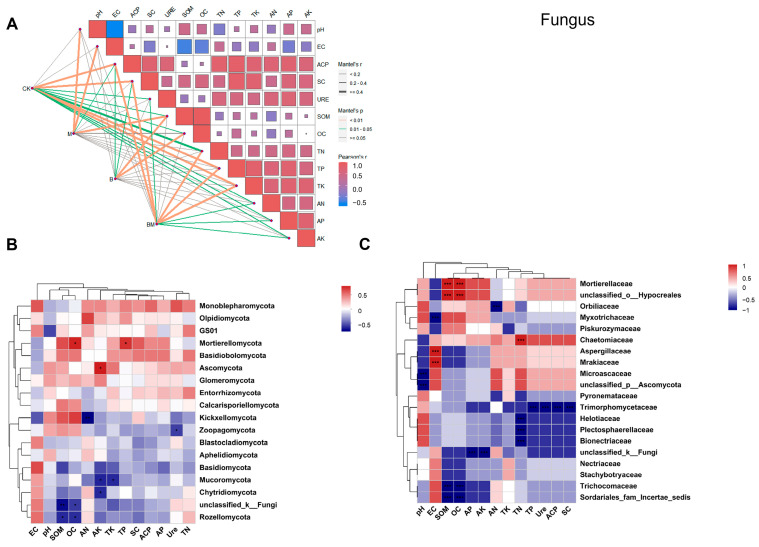
Relationships between the rhizosphere fungal populations under various treatments and soil enzyme activity, as well as interactions among soil physicochemical parameters (**A**). Spearman correlation analyses for the physicochemical factors and the top 20 abundant fungi at the phylum (**B**) and family (**C**) levels. Stars represent significant correlations (*p* < 0.01 for two stars, *p* < 0.05 for one star and *p* < 0.001 for three stars) of these indexes. Color brightness indicates correlation coefficient values.

In terms of rhizosphere fungal communities, the treatments resulted in varying relationships between the rhizosphere fungal communities and the physicochemical parameters of the soil (Figure 8A). There were notable relationships between the rhizosphere fungal populations and soil nutrients and soil enzyme activity across all treatments. At the phylum level of fungi, *Mortierellomycota* showed significant correlations with TP and OC. Moreover, highly significant and negative correlations were exhibited between *Kickxellomycota* and AN and SOM, and between *Pozellomycota* and OC (Figure 8B). At the family level of fungi, among the fungi exhibiting relatively increased abundance, *Mortierellaceae* showed positive and highly significant correlations with SOM and OC, but *Trichocomaceae* exhibited negative and highly significant correlations with them. *Helotiaceae*, *Plectosphaerellaceae,* and *Bionectriaceae* showed significant and negative correlations with TN, but *Chaetomiaceae* exhibited significant and positive correlations with it. Finally, *Trimorphomycetaceae* showed highly significant and negative correlations with TP, URE, ACP, and SC (Figure 8C). These results clearly indicate that soil physicochemical properties remarkably affected microbial community structure under different treatments.

## 3. Discussion

In this study, the bacterial strain N33 adhered well to the biochar, and the colonization of N33 in rhizospheric soil under BM treatment was higher than that under M treatment. This may be an outcome of the porous structures and coarse surface of the biochar facilitating N33’s attachment and proliferation (Figure S1). Similar studies showed that because of its rough surface and porous structure, maize biochar was successfully loaded with the strain *Pseudomonas* sp. NT-2 [24]. Sun et al. found that VOCs absorbed to biochar could enhance *B. mucilaginosus*’s survival, potentiating biochar as a microbial carrier for producing inoculants [25]. Therefore, the dominant substances on the surface of biochar are available for microbes, leading to a series of reactions and influencing biochar loading with microbes.

Based on the data on pecan plant growth, the biomass, seeding growth, stem diameter, and leaf area of plants under BM treatment were remarkably enhanced. These data indicated that biochar loaded with bacteria positively affected the growth and development of plants. In addition, we discovered that the nutrient indexes of pecan plants under BM treatment were significantly increased. Similar outcomes were reported by An Shi, who found that biochar loaded with bacteria could alleviate metal toxicity towards *S. alfredii* through a decrease in oxidative damage and enhanced chlorophyll content, ultimately facilitating the growth of the plant [26]. The leaf area and chlorophyll contents of plants under BM treatment were significantly higher than those in the control group. As the major organ of respiration and photosynthesis, leaves are extremely critical for plant growth. The leaf area of soybean plants was closely related to photosynthetic parameters, especially for transpiration rate (E), stomatal conductance (Gs), and photosynthetic rate (Pn) [27]. Moreover, the chlorophyll content was strongly correlated with photosynthesis, representing the efficiency of photosynthesis [28]. The chlorophyll content in tomato plants after treatment with biochar loaded with bacteria was found to increase by 1.1–1.4 times after 30 d compared with the control [29]. In our study, the contents of soluble sugar and proteins in pecan plants were higher under BM treatment. Energy and metabolic intermediates, as crucial plant hormone regulators, are obtained from soluble sugar for the growth and development of the plant [30].

In an earlier study, the combination treatment of endophytes and biochar in the soil of soybean plants stimulated nutrient uptake, causing enhanced nutrient (e.g., sugar) accumulation in the soybean plants [31]. Therefore, biochar and bacteria may work synergistically to produce beneficial effects on plants. In our study, soil EC decreased following the application of biochar, bacteria, and biochar loaded with bacteria. Similar research demonstrated that the decreased soil EC may be related to the dissolution of soil salt by biochar [32]. Additionally, the decrease in soil EC post-bacterial application may be due to microbe metabolism that breaks down some of the salt [33]. SOM content is extremely crucial for soil remediation and its water resource utilization [34]. In this study, compared with other treatments, the content of SOM under BM treatment was significantly increased. This result showed that a portion of the organic matter in the biomass was retained during the formation of biochar [35]. N, P, and K, essential elements for plant metabolism, can be directly absorbed by plant roots [36]. In this study, we found that the soil total nutrient indexes and available nutrient indexes under BM treatment were increased. Similarly, the total available P and N in soil were reported to be increased after the application of biochar loaded with bacteria [37]. Moreover, biochar loaded with SL-44 could improve photosynthetic capacity and endogenous phytohormone biosynthesis, causing high uptake of TK, TP, and TN [38]. In addition, soil enzyme activity is a vital index for assessing soil fertility. In this study, we found noticeably higher soil saccharase and neutral phosphatase contents in the BM-treated group than in the control group. According to Jabborova et al., applying PGPR and biochar together greatly increased the enzymatic activities and the amount of nutrients in the soil, which in turn helped to boost soybean growth and yield [39]. In conclusion, this study shows that applying BM treatment can promote soil enzyme activity and improve the soil’s chemical and physical characteristics, hence promoting pecan development.

The microbial population in the soil influences plant development and nutrient uptake. In this study, both M and BM treatments remarkably broadened the soil bacterial community diversity. The *Firmicutes* abundance in rhizospheric soil was substantially elevated under BM and M treatments compared with the control and B-treated groups. The *Proteobacteria* abundance under all the other treatments decreased compared with the control treatment. This could be a result of how soil pH influences nutrient (small molecules, P, and N) solubilization, which in turn influences the development of microbes [40]. Relative to the control, the *Bacillaceae* abundance in soil substantially differed under M and BM treatments. Based on this result, we supposed that biochar could elevate *Bacillaceae* abundance in soil and facilitate their colonization of crop roots [41]. At the phylum level of fungi, the fungal community structure was simplified after BM treatment, indicating that BM treatment possibly renders broad-spectrum disease resistance as plenty of fungi species are opportunistic phytopathogens. Similar research has indicated that the use of bacterial suspensions caused improvements in soil properties and nutrients, thereby decreasing the fungal community diversity [42]. This result was in agreement with our study. At the family level of fungi, both M and BM treatments enhanced the relative *Mortierellaceae* abundance, in comparison with the CK. *Mortierellaceae* species were among the most often encountered soil fungi globally [43]. Based on the correlation heatmaps, we found that the relatively high-abundance bacteria were negatively correlated with EC and pH and positively correlated with nutrient contents. This result was similar to the study conducted by Wenqiang Fan [44]. Moreover, both fungi and bacteria were significantly associated with AN, AK, AP, and pH. AN and AK were reported as the primary factors of the soil related to bacterial and fungal community shifts [45]. Overall, our results suggest that biochar loaded with N33 could promote beneficial microbial enrichment in the rhizosphere of plants, which is possibly responsible for promoting plant growth.

## 4. Materials and Methods

### 4.1. Bacterial Strains and Cultivation

The *Priestia aryabhattai* strain N33 was isolated from the rhizospheric soil of pecan plants and stored at −80 °C in our laboratory. We generated streptomycin-resistant N33 (hereafter designated as N33) by the transformation of a streptomycin-resistance gene. N33 was streaked on solid medium, single colonies were picked and added into an approximate volume of nutrient broth (NB) (10 g L^−1^ peptone (Qingdao Hope Bio-Technology Co., Ltd., Qingdao, China), 5 g L^−1^ NaCl, (pH 7.0–7.2), 3 g L^−1^ beef extract), and cultivated at 30 °C under 170 rpm until approximate concentrations of the cell suspension were obtained.

The original N33 was streaked on NA plates, and a mono-colony was picked and transferred to NB medium containing 1 μg/mL streptomycin. The inoculum was placed in a shake incubator under 28 °C for 24 h, then streaked on NA plates (1 μg/mL) again. Next, a mono-colony was picked and transferred to NB medium containing 5 μg/mL streptomycin for the next round and streaked on NA plates (5 μg/mL) again. In this way, mono-colonies were successively picked and transferred to NB medium containing 10, 50, and 100 μg/mL streptomycin, until the concentration of streptomycin ultimately reached 500 μg/mL. The mutant whose colony morphology and PGP characteristics were similar to the original strain was selected in this study.

### 4.2. Preparing Biochar Loaded with N33

Pecan seed shells were the source of the biochar used in the current study. The dried pecan seed shells were pyrolyzed at 500 °C for 2 h in a muffle furnace without oxygen. Pyrolyzed materials were ground and passed through a 0.15 mm sieve, sterilized, and oven-dried at 80 °C. The resultant biochar was stored in a glass container until utilized.

N33 was cultivated in 200 mL NB at 30 °C in a flask, shaken at 170 rpm, for one full day. The mixture was centrifuged at 4 °C and 6000 rpm for 30 min. The precipitates were then washed with sterile 0.85% NaCl solution three times and suspended in the same solution of NaCl in an appropriate volume, and the OD_600_ of cell suspensions was determined. Finally, cell suspensions were adjusted to OD_600_ = 0.8, 0.4, 0.16, 0.08, and 0.04 to obtain the required N33 bacterial suspensions [46].

Loading N33 onto biochar was conducted as follows: the biochar and N33 suspensions of different concentrations were mixed at a 1:5 (*m*:*v*) ratio and shaken at 170 rpm for 24 h at 30 °C, followed by freeze-drying in a vacuum oven. Following 24 h drying, biochar loaded with N33 (biochar with microbes, BM) was obtained. SEM was utilized for biochar and BM microscopic structure observation.

### 4.3. Measurement of the Loaded Number and Survival Rate of N33

First, 1 g BM was adequately mixed with 9 mL ddH_2_O and centrifuged at 1000 rpm. Then, the supernatant was aspirated and inoculated on the NA plate and cultivated at 28 °C for 24–48 h to determine the bacterial number. The number of bacteria loaded on biochar can be calculated using the following formula:

Number of loaded bacteria (CFU/mL) = N1 − N2 (N1, the total bacterial number in the initial bacterial suspensions; N2, the bacterial number in the supernatant after loading).

The survival of N33 was assessed by adding 1 g of N33-loaded biochar to 9 mL of sterile distilled water and adequately vortexing the mixture to ascertain absolute bacterial separation from the biochar. Gradient dilutions of bacteria in the distilled water were inoculated on NA plates to count the number of colonies, and each treatment was repeated three times. Then, the plates were cultivated at 28 °C for 24–48 h, and the number of colonies was counted and recorded [35].

Number of biochar-supported bacteria (CFU/mL) = A × R (A, number of colonies on plates; R, dilution ratio)

Survival rate of N33 (%) = Number of biochar-supported bacteria/Number of biochar-loaded bacteria × 100.

### 4.4. Pot Assays

Pot assays were conducted to evaluate the effect of N33 (microbe, M), biochar (B), and the biochar loaded with N33 (biochar with microbes, BM) application on pecan plants. Nonwoven bags (25 cm × 35 cm) were filled with 8.5 kg field soil. The original field soil physicochemical attributes are summarized in Table S1. Four treatments were carried out: (a) CK (routine management), (b) M (routine management with 50 mL N33 suspension (8.32 × 10^5^ CFU/mL)), (c) B (routine management with 10 g biochar), (d) BM (routine management with 60 g BM); the number of bacteria and weight of biochar in BM were consistent with those in B and M. N33 suspensions were poured along the roots of each seedling. Biochar and BM were added to the soil and mixed before transplanting. Each treatment contained five replicates and one seedling was planted in each pot. The entire assay was repeated three times.

### 4.5. Colonization of N33 in Soil

Rhizospheric soil samples of three pecan seedlings in each treatment were collected 15 days after M and BM treatments. After shaking off the large clods attached to the roots, the rhizospheric soil 2 mm from the surface of the root system was gently brushed off with a brush and collected. One gram of rhizospheric soil was mixed in 10 mL of sterile water and thoroughly shaken for 30 min. Serial dilutions of the samples were incubated on NA plates supplemented with streptomycin, and the colonies were counted [47,48].

### 4.6. Determining Plants’ Biochemical and Physiological Characteristics

Plants were harvested five months after transplanting, the fresh weight and plant height were recorded, and the samples were rinsed with sterile deionized water. Subsequently, the plants were placed into an oven (105 °C, 15 min) at 60 °C for drying to a constant weight, and the constant dry weight was recorded.

Fresh leaves (0.1 g) were collected for homogenization in phosphate buffer (pH 7.8, 4 °C). The centrifuged (12,000 rpm, 20 min) homogenates were utilized for enzymatic assays. The root vigor of seedlings was assessed utilizing the triphenyl tetrazolium chloride (TTC) reduction method. Superoxide dismutase (SOD) activity was measured at 560 nm [49] using a Microplate Reader (Bio Tek, Fenton, MO, USA), and peroxidase (POD) activity was measured at 470 nm [50]. The contents of soluble sugar were estimated by the anthrone colorimetry method, and the soluble proteins were estimated by the Coomassie brilliant blue method [51]. Fresh leaves (0.1 g) were extracted in absolute ethyl alcohol. The absorbance of the obtained extract was determined at 660, 665, and 649 nm for chlorophyll a and b and carotenoids, respectively, utilizing a microplate reader [52].

Determination of total potassium (TK), total phosphorus (TP), and total nitrogen (TN) was conducted according to the methods described by Shan et al. [53,54,55].

### 4.7. Measurement of Soil Enzyme Activities and Chemical Properties

Soil pH was measured in a 1:5 soil/water suspension and the conductivity of soil was measured by a conductivity meter. The content of soil organic matter (SOM) was estimated by the potassium dichromate oxidation method. TN in soil samples was estimated by the Kjeldahl method, TP by spectrophotometry, and TK by flame spectrophotometry and melting with NaOH. The content of available nitrogen (AN) was determined by acid–base titration, the molybdenum blue colorimetric protocol was employed to determine available phosphorus (AP), and the flame photometric approach (BWB XP, BWB Technologies, UK) was used for the determination of quick-acting potassium (AK).

Soil samples were incubated in urea (10%) and citrate buffer (pH 6.7) for 24 h at 37 °C, and the urease activity was estimated at 578 nm employing a microplate reader [56]. Soil saccharase was determined by sodium thiosulfate titration. Soil neutral phosphatase (S-NP) activity was estimated in phenyl phosphate-incubated soil at 510 nm employing a microplate reader [57].

### 4.8. Community Analyses of Soil Fungi and Bacteria

The total DNA was extracted from the collected rhizospheric soil by the Majorbio Genomics Institute (https://www.majorbio.com, accessed on 1 December 2023) for *16S rRNA* gene and ITS region high-throughput sequencing based on the Illumina Mi-Seq platform. Relative bacterial and fungal abundances were determined by the Majorbio Cloud Platform (www.majorbio.com, accessed on 1 December 2023). The visual analyses were performed using the R (4.3.2) package “ggplot2” (0.9.2).

### 4.9. Statistical Analysis

IBM SPSS 27 and Excel 2019 were used to statistically evaluate all of this experiment’s data. Determination of statistical significance was carried out by an analysis of variance followed by multiple comparisons employing Tukey’s test; a *p*-value < 0.05 was deemed statistically significant. Graphs were visualized using Origin 2022. Microbial and soil index correlations were plotted using the “ggplot2” package in R 4.3.2.

## 5. Conclusions

In this study, biochar could serve as a carrier for N33. The application of biochar loaded with N33 could improve the growth of pecan plants and the soil environment, thus promoting nutrient uptake in the plants. Furthermore, the diversity of microbial communities was increased significantly, such as the relative abundance of Firmicutes and Chloroflexi at the phylum level, and Bacillaceae and Paenibacillaceae at the family level. In light of this, the combination of biochar and functional bacteria is feasible for both plants and soil.

## Figures and Tables

**Table 1:** The screening of the most suitable N33 concentration.

Parameters	CK	M1	M2	M3	M4	M5
Number of bacteria in the initial suspensions (10^5^ CFU/mL)	0	6.68 ± 0.62	4.58 ± 0.52	2.18 ± 0.3	1.3 ± 0.04	2.5 ± 0.09
Number of bacteria in the supernatant (10^5^ CFU/mL)	0	3.35 ± 0.25	1.93 ± 0.32	1.3 ± 0.04	2.83 ± 0.07	0.06 ± 0.07
Number of bacteria loaded on biochar (10^5^ CFU/mL)	0	3.33 ± 0.4	2.65 ± 0.56	0.88 ± 0.32	1.02 ± 0.03	1.83 ± 0.03
Number of biochar-supported bacteria (10^5^ CFU/mL)	0	2.83 ± 0.48	1.6 ± 0.28	0.43 ± 0.14	0.35 ± 0.08	0.67 ± 0.03
Survival rate (%)	0	84.6 ± 0.04	60.8 ± 0.03	49.49 ± 0.03	34.16 ± 0.05	36.11 ± 0.11
pH	6.69 ± 0.09	7.03 ± 0.09	7.29 ± 0.12	7.2 ± 0.14	7.24 ± 0.06	7.22 ± 0.08
EC (ms/cm)	12.29 ± 0.03	9.23 ± 0.05	9.67 ± 0.53	12.11 ± 0.28	12.27 ± 0.12	11.23 ± 1.08

**Table 2:** Effects of different treatments on root vigor and POD and SOD activities in pecan plants.

Group	Root Vigor (mg TTF·(g·h)^−1^)	POD Activity (U·g^−1^)	SOD Activity (U·g^−1^)
CK	11.13 ± 2.029 b	73.33 ± 29.78 a	110.63 ± 20.87 a
M	9.93 ± 2.65 b	65 ± 20.49 a	131.41 ± 29.35 a
B	9.84 ± 4.72 b	63.33 ± 24.63 a	94.72 ± 9.6 a
BM	19.52 ± 3.56 a	30 ± 4.47 a	141.65 ± 33.7 a

Notes: CK: control. The data are the means ± standard deviation of three replicates; different lowercase letters indicate significant differences at *p* < 0.05.

## Data Availability

All datasets for this study are included in the manuscript and/or the Supplementary Files. All data that support the findings of this study are available from the corresponding authors upon reasonable request.

## Supplementary Materials

The following supporting information can be downloaded at: https://www.mdpi.com/article/10.3390/plants13091226/s1, Figure S1: The scanning electron microscope (SEM) images and the amount of N33 in soil; Table S1: The physicochemical properties of the original field soil.
