# Supplementary figures and images for: Biochar Loaded with a Bacterial Strain N33 Facilitates Pecan Seedling Growth and Shapes Rhizosphere Microbial Community
Source: Plants (Basel). 2024 Apr 28;13(9):1226. doi: 10.3390/plants13091226 (PMC11085327; doi:10.3390/plants13091226)

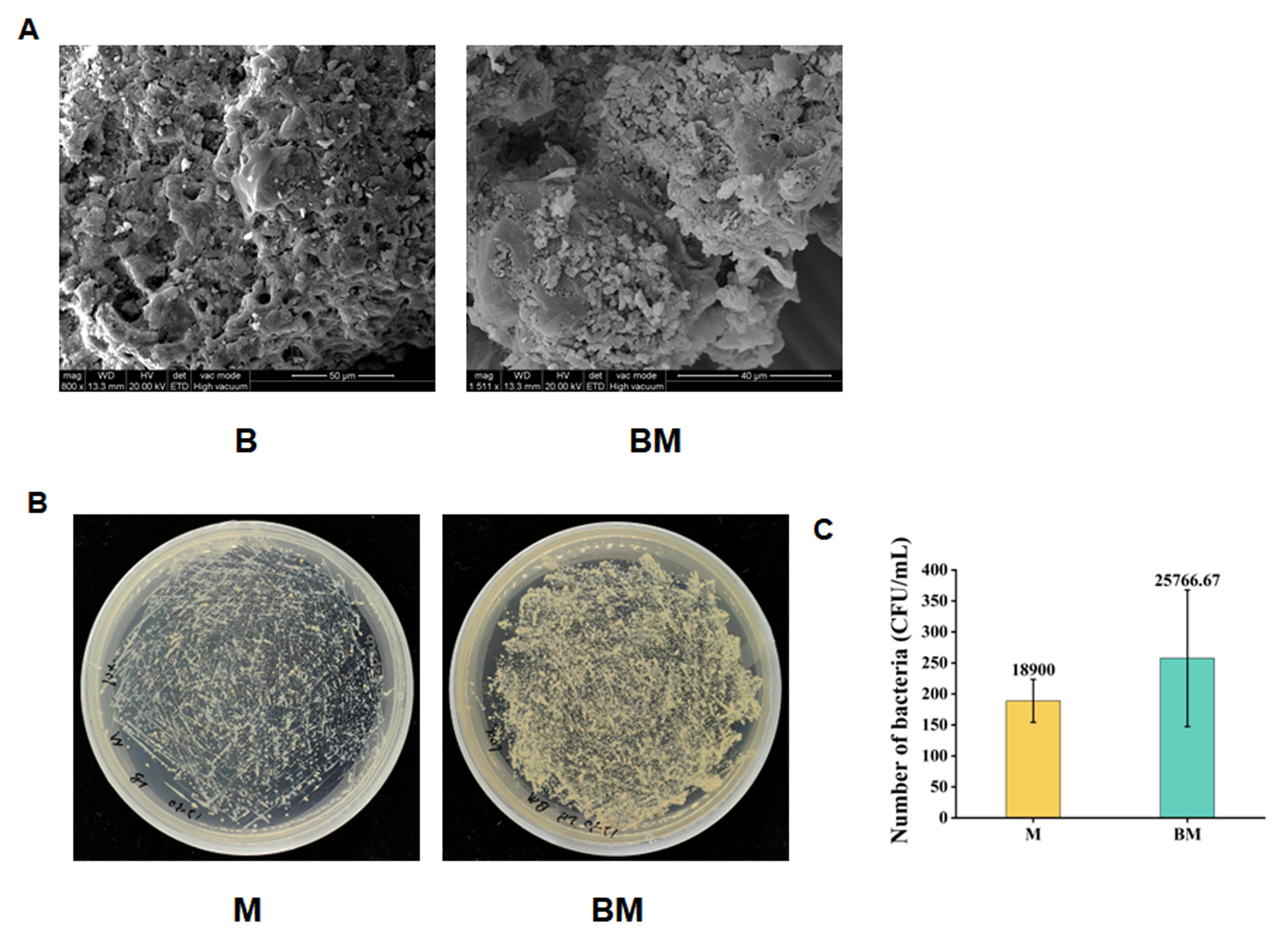

Supplement: Supplementary file 1 [file plants-13-01226-s001.zip › Figure S1.jpg]
